# Long-Term Outcomes of Transvenous Lead Extraction: A Comparison in Patients with or without Infection from the Italian Region with the Oldest Population

**DOI:** 10.3390/jcm12134543

**Published:** 2023-07-07

**Authors:** Luca Barca, Giuseppe Mascia, Paolo Di Donna, Paolo Sartori, Daniele Bianco, Roberta Della Bona, Stefano Benenati, Andrea Carlo Merlo, Antonia Luisa Buongiorno, Niki Kaufman, Antonio Vena, Matteo Bassetti, Italo Porto

**Affiliations:** 1 Department of Internal Medicine, University of Genoa,16132 Genoa, Italy; lucabarca1993@gmail.com (L.B.); stefanobenenatimd@gmail.com (S.B.); andreacarlo.merlo@gmail.com (A.C.M.); antonialuisabuongiorno@gmail.com (A.L.B.); niki_net12@yahoo.com (N.K.); italo.porto@gmail.com (I.P.); 2 Cardiovascular Disease Unit, IRCCS Ospedale Policlinico San Martino, 16132 Genova, Italy; didonnapaolo11@gmail.com (P.D.D.); dott.paolosartori@gmail.com (P.S.); danielebianco85@hotmail.com (D.B.); roberta.dellabona@gmail.com (R.D.B.); 3 Infectious Disease Clinic, Department of Health Sciences, IRCCS Ospedale Policlinico San Martino, University of Genoa, 16132 Genoa, Italy; anton.vena@gmail.com (A.V.); matteo.bassetti@hsanmartino.it (M.B.)

**Keywords:** lead extraction, device infection, lead malfunction, aging population

## Abstract

Background: The gold standard for the treatment of cardiac implantable electronic devices (CIEDs)-related infection and lead malfunction is transvenous lead extraction (TLE). To date, the risk of mortality directly related to TLE procedures is relatively low, but data on post-procedural and long-term mortality are limited, even more in the aging population. Methods: Consecutive patients with CIEDs who underwent TLE were retrospectively studied. The primary outcome was the endpoint of death, considering independent predictors of long-term clinical outcomes in the TLE aging population comparing patients with and without infection. Results: One hundred nineteen patients (male 77%; median age 76 years) were included in the analysis. Eighty-two patients (69%) documented infection, and thirty-seven (31%) were extracted for a different reason. Infected patients were older (80 vs. 68 years, *p*-value > 0.001) with more implanted catheters (*p*-value < 0.001). At the last follow-up (FU) available (median FU 4.1 years), mortality reached 37% of the patient population, showing a statistically significant difference between infected versus non-infected groups. At univariable analysis, age at TLE, atrial fibrillation, and anemia remained significant correlates of mortality; at multivariable analysis, only patients with anemia and atrial fibrillation have a 2.3-fold (HR 2.34; CI 1.16–4.75) and a 2.5-fold (HR 2.46; CI 1.33–4.54) increased rate of death, respectively. Conclusion: Our long-term data showed that aging patients who underwent TLE for CIED-related infection exhibit a high mortality risk during a long-term follow-up, potentially leading to a rapid and effective procedural approach in this patient population.

## 1. Introduction

The implant rate of cardiac implantable electronic devices (CIEDs) has increased progressively due to increasing life expectancy [1,2,3,4,5,6,7,8], and, more commonly today, CIEDs are implanted in older patients with many comorbidities [7,8,9]. Advanced age is related to multiple comorbidities and frailty, potentially increasing the probability of complications during invasive procedures [9,10]. Although infrequent, CIED-related infections, as well as lead malfunction, represent a serious complication after cardiac device implantation [5], with several data showing device complications associated with significant mortality and morbidity [7,8,9]. Transvenous lead extraction (TLE) represents the gold standard for the treatment of CIED-related infection and lead malfunction [8]. To date, the mortality risk directly related to TLE procedures is relatively low [6,7,8], while data regarding post-procedural and long-term mortality are limited [8,9,11]. Considering the aging population, TLE will play an increasing role in the future management of these subjects. Therefore, in our study, we analyze independent predictors of long-term clinical outcomes of patients undergoing TLE, assessing the prognostic role of an infective indication on long-term survival in the aging population.

## 2. Methods

We identified a cohort of 119 consecutive patients undergoing TLE at our institution in the Liguria region. Liguria is an Italian region located in the northwest part of Italy, and it is currently the oldest Italian Region [12]. We retrospectively analyzed patient characteristics, procedural indications, and clinical outcomes. For the purpose of the study, the patient population was categorized as infected and non-infected, following the Heart Rhythm Society (HRS) consensus document on TLE [10] and European Heart Rhythm Association (EHRA) expert consensus statement on lead extraction [13]. An infective indication included a systemic (bacteremia and/or endocarditis) or local (pocket infection or erosion) infection, while a non-infective indication included lead malfunction and venous thrombosis. The primary endpoint of the study was a comparison of long-term mortality between patients with or without infection after hospital discharge. Secondary endpoints included complete procedural success, procedural failure, and the occurrence of complications defined by the HRS and EHRA consensus [10,13]. For the aim of the study, we considered complications as only the adverse events occurring before hospital discharge. In particular: death, cardiac tamponade, cardiac/vascular avulsion or tear, respiratory arrest, pulmonary embolism, and stroke were regarded as major complications, whereas complications that did not meet the major criteria were considered minor complications.

### 2.1. Extraction Techniques

After obtaining written informed consent, invasive hemodynamic monitoring through an arterial line was placed. Procedures were performed under general anesthesia and cardiac surgical backup. Device removal and disconnection of the lead(s) were performed through an infraclavicular incision. The lead(s) were extracted through a subclavian approach. Lead removal with simple traction was attempted as the first step. If lead removal proved unsuccessful, the lead was cut; a locking stylet (Liberator Cook Medical) was introduced, and traction was reattempted. If this still proved unsuccessful at the “first step”, a mechanical sheath was used, eventually considering a powered sheath (Evolution Cook Medical) when necessary. Laser-assisted lead extraction was never performed in our center. Complete procedural success was achieved if all targeted leads/lead material were removed from the vascular space, while clinical success was achieved if all targeted leads/lead material were removed with retention of a small lead portion (<5 cm), with no impact on the outcome goals. In cases of infection, complete removal of both foreign material and infected tissue was mandatorily performed. Failure was considered if neither complete procedural success nor clinical procedural success was achieved.

### 2.2. Antibiotic Therapy

In patients with device infection, an empiric antibiotic therapy such as daptomycin (i.v. 8–10 mg/kg every 24 h) or vancomycin (i.v. 30–60 mg/kg/day) until a potential microbiological identification was performed according to the clinical scenarios. Cefepime (i.v. 2 g every 8 h) or ceftriaxone (i.v. 2 g every 24 h) or gentamycin (i.v. 5–7 mg/kg every 24 h) were only considered in case of systemic symptoms [14]. In patients with systemic infection, once the pathogen was identified (usually within 3 days), the antibiotic treatment was tailored to the antimicrobial susceptibility pattern. In this scenario, the collaboration between cardiologists and infectious disease specialists with expertise in the field of CIED-related infection was of primary importance. The duration of therapy could depend on the presence or lack thereof of concomitant systemic infection and could vary from 2 weeks in case of isolated pocket infection to typically 4–6 weeks in case of positive blood cultures and or vegetations. In particular, all patients with systemic infection underwent appropriate antibiotic treatment after removal according to antibiograms of positive bacteria cultures and current guidelines [14].

### 2.3. Statistical Analysis

Continuous variables were expressed as mean ± standard deviation or median (interquartile range) and compared with the Student T test or Mann–Whitney test, as appropriate. Categorical variables were expressed as frequencies and percentages and compared with the Chi-square or Fisher exact test, as appropriate. Time-to-event curves were built, and survival was compared between infected and non-infected patients using the log-rank test. Univariable and multivariable Cox analyses were carried out to explore the predictors of survival, deriving hazard ratios (HR), and associated 95% confidence intervals. Candidate variables were entered in the multivariate analysis when proven to be significant univariate predictors. All tests were 2-tailed, and *p* < 0.05 was considered significant. Statistical analysis was performed using “R” software (the R foundation for statistical computing version 3.6.2. using the “meta” package).

## 3. Results

**Figure 1:**
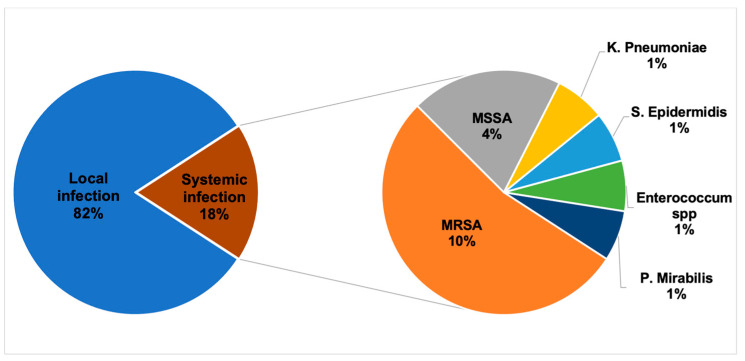
Microbiology of transvenous lead extraction from 82 patients with infection. MSSA = Meticillin-Sensitive *Staphylococcus aureus*, MRSA *=* Methicillin-resistant *Staphylococcus aureus*.

Between January 2014 and April 2020, 119 patients (224 leads) underwent TLE, out of which 82 patients (69%) had an infection diagnosis (181 leads). Males represented 77% of patients, and the median age at the TLE procedure was 76 (67–82) years. Table 1 shows the baseline characteristics of the patient population. Infected patients were older (80 vs. 68 years, *p*-value > 0.001), with more implanted catheters (*p*-value < 0.001) despite a lower incidence of heart failure (43.4% versus 65.7%, *p*-value = 0.03), whereas other comorbidities were balanced compared to non-infected patients. The median time from first device implantation to TLE was longer in the infected population (109 months versus 66 months, *p* = 0.03) compared to non-infected patients. Table 2 shows a comparison between infected and non-infected patients. In the infection-related group (82 patients), pathogenic organisms were identified in 18% of cases: positive microbiologic culture results showed Gram-positive in 15% of cases, and *Staphylococcus aureus* was the most commonly detected bacterium, as shown in Figure 1. Moreover, we compared the characteristics of patients with local infection versus systemic infection, documenting no significant difference among baseline characteristics (See Table S1 from Supplementary Materials). Among TLE procedures, a total of 224 leads were extracted, with a mean of 1.9 ± 0.9 lead per procedure. The mean procedural time was 129 ± 50.2 min; the oldest lead was in place for 396 months. Complete procedural success was achieved in 84.9% of patients, with a 91.6% clinical success rate. In total, 194 leads (81.1%) were removed completely, 16 leads (6.7%) were removed with retention of a small portion of lead without negatively affecting outcome goals and therefore leading to clinical success, one lead (0.42%) was submitted to surgical lead extraction, and one lead (0.42%) was considered as failure. Procedural characteristics are reported in Table 3.

### 3.1. Procedural Complications

A single case (0.84%) of death was documented in the subject with an indication of lead malfunction due to cardiac avulsion during the procedure. Surgical extraction was required in three cases after cardiac tamponade. In a fourth case, initially performed for lead malfunction, the procedure failed because of a lead fracture at the level of the left subclavian. A total of seven intraprocedural complications occurred, including two strokes.

### 3.2. Short-Term Outcome

Ten patients (8.4%) died at the hospital (25-days average after TLE), six of whom individuals had infectious indications for TLE (three local and three systemic). The three patients with systemic infection died of multiorgan failure secondary to sepsis due to methicillin-resistant *Staphylococcus aureus*, *P. aeruginosa* or *K. pneumoniae*. Four patients with no infection died at short-term follow-up: two patients died in the hospital due to progressive heart failure 15 days after TLE, one patient died of a complication of a renal biopsy performed during hospitalization (exsanguinating retroperitoneal hemorrhage), last patient at 85 years old died due to spontaneous cerebral hemorrhage 1 week after TLE.

### 3.3. Long-Term Outcome

**Figure 2:**
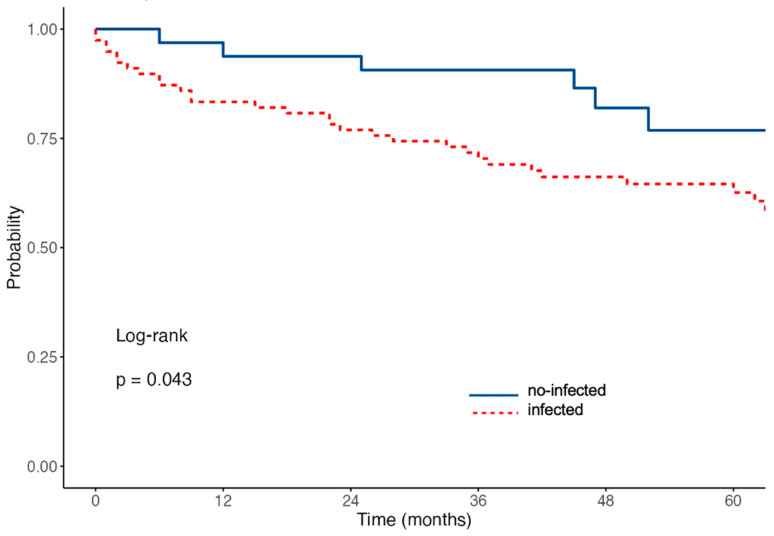
Kaplan Meier analysis of all-cause death after hospital discharge.

At the last follow-up available (median observation time 49 months, range: 1–93 months), mortality reached 37% of the patient population, including only patients after hospital discharge. The mortality analysis ended on January 2023: reasons for death during long-term outcomes were not available for most patients. Kaplan Meier curves describing mortality after hospital discharge of the TLE population showed a statistically significant difference between infected versus non-infected groups (Figure 2).

### 3.4. Predictors of Mortality

Univariable and multivariable analyses by Cox regression identified several correlates of mortality (Table 4). At univariable analysis, age at TLE, atrial fibrillation, and anemia remained significant correlates of mortality. In particular, the instantaneous mortality rate increases by 3% per year of patient age (HR 1.03; CI 1.01–1.06). At multivariable analysis, patients with anemia and atrial fibrillation have a 2.3-fold (HR 2.34; CI 1.16–4.75) and a 2.5-fold (HR 2.46; CI 1.33–4.54) increased rate of death, respectively.

## 4. Discussion

This study analyzes long-term mortality in TLE procedures from a medium-volume single center in the oldest Italian Region: not by chance, the patients are older compared to other large studies such as LExlCon [15], ELECTRa [16], and PROMET [17] (mean age 76 versus 63–65 years, respectively), while the proportion of males is comparable to the aforementioned studies (79.3%) [15,16,17]. In the results, long-term mortality is significantly higher in the older median-age CIED-infected population when compared to the non-infected population; actually, infection-related indications were different when compared to larger studies (68.9% in our study versus 46–57%) [15,16,17]. This finding may be because of the lower threshold for performing TLE in non-infected CIEDs, due to a potentially higher procedural risk in the older population since octogenarians are deemed as high-risk candidates for TLE; despite in previous little populations, the old age could not influence TLE effectiveness, being successfully performed [18,19].

The procedural success rate was achieved in 91.6%, a slightly lower percentage than the studies mentioned above (94.3–98.7%) [15,16,17] without any significant difference when comparing CIED-infected and non-infected populations. Intraprocedural mortality was low (0.84%) and comparable to large series: the ELECTRa registry [16] showed a procedural mortality of 0.5%, while Wazni O et al. [15] showed a procedural mortality of 0.28%. On the other hand, in-hospital mortality was 8.4%: older age and overlapping comorbidities could increase the risk in patients requiring TLE. According to a prospective multicenter study [16], age over 68 years is a predictor of increased all-cause mortality during hospitalization. Finally, the results show that long-term mortality is significantly higher in the older median-age CIED population, documenting an all-cause mortality rate of 37% during the entire follow-up period. Long-term mortality after TLE is significantly higher in patients with infection; notably, the survival curves of patients undergoing TLE for infection diverge from those of patients undergoing TLE for lead malfunction or other indications from the first few months after hospital discharge. These findings are consistent with a recent report from Arabia et al. [20], documenting that patients who perform TLE for CIED-related infection may exhibit a 30% mortality rate during a 6.5 median follow-up. Migliore et al. [18] recently described long-term mortality in elderly patients undergoing TLE: the main indication for TLE was an infection in 84.3% of cases with an overall mortality rate of 29% during a mean follow-up of ≈2 years. Finally, Henrikson et al. [21] described midterm mortality in a small population undergoing TLE for infectious indications, documenting a 30% mortality rate during the follow-up.

In our results, anemia and atrial fibrillation were the strongest correlates of mortality in multivariable analysis, and age at the extraction reached statistical significance. Therefore, in an older population undergoing TLE, more effort should be dedicated to the preoperative e postoperative treatment of comorbidities such as severe anemia and poorly managed atrial fibrillation. Moreover, considering that survival continues to be burdened by the progression of multiple chronic diseases beyond the clinical resolution of the infection, old patients who undergo successful TLE (especially for an infectious cause) remain at high risk of death at a median follow-up of 49 months. Also, infection prevention may have a significant impact on long-term mortality reduction. In particular, a preoperative antibiotic strategy combined with an early procedural approach is extremely important in order to have the best clinical condition at baseline and potentially a more favorable prognosis, also in older populations. Not by chance, today’s recommendations suggest complete device and lead removal for all patients with CIED infection [10].

## 5. Limitations

In terms of limitations, this study is a retrospective analysis and thus is subject to bias. The main limitation is the small number of patients (n: 119), which limits data analysis. The cohort was limited to a single, medium-volume academic center, and the experience may differ at other types of institutions. In addition, the details regarding the mode of death are not available.

## 6. Conclusions

This study evaluates the long-term outcomes of TLE in elderly patients with or without infection from a single-center experience. Our data show that patients undergoing TLE for CIED-related infection have a high risk of mortality during a long-term follow-up, potentially leading to a rapid and effective procedural approach in this patient population.

## Figures and Tables

**Table 1:** Overall study population characteristics.

Variable	n = 119 (%)
Male sex	92 (77.3%)
Coronary artery disease	40 (36.4%)
Heart failure	56 (50.5%)
Atrial fibrillation	40 (36.4%)
Systemic arterial hypertension	74 (67.3%)
Diabetes	24 (21.8%)
Hemoglobin, g/dL	12.3 ± 2.0.
Anemia Men (<13.5 g/dL under 70; <12 g/dL over 70) Women (<11.5 g/dL)	24 (22%)
White blood cells, 10^9^/L <4500 (leukopenia) 4500–9800 (normal range) >9800 (leukocytosis)	37 (34.3%) 60 (55.6%) 11 (10.2%)
C-reactive protein ≥3 mg/dL	71 (72.5%)
Creatinine, mg/dL	1.1 (0.5)
Chronic kidney disease (men: ≥1.4 mg/dL, women: ≥1.2 mg/dL)	31 (28.7%)
Positive blood cultures *Methicillin-Resistant-Staphylococcus aureus* *Methicillin-Susceptible-Staphylococcus aureus* *K. pneumoniae* *S. epidermidis* *E. faecalis, E. faecium* P. mirabilis * out of 82 pts diagnosed with infection	15 (18.3%) * 8 3 1 1 1 1
Left ventricular ejection fraction <30% 30–50% >50%	16 (18.4%) 39 (44.8%) 32 (36.8%)
Age at extraction, years	76.4 (15.4)
Infection local systemic * out of 82 pts diagnosed with infection	67 (82%) * 15 (18.3%) *
Number of implants 1 2 3 4	46 (60.5%) 19 (25%) 9 (11.8%) 2 (2.6%)
Time from to catheters, months	84.5 (85)
Type of device Single pacemaker (PM) Single Implantable cardioverter-defibrillator (ICD) Dual PM Dual ICD Cardiac resynchronization therapy pacemaker (CRT-P) Cardiac resynchronization therapy defibrillator (CRT-D)	4 (3.5%) 22 (19.1%) 42 (36.5%) 15 (13%) 12 (10.4%) 20 (17.4%)
Coil Single Dual * out of 58 pts implanted with single ICD, dual ICD, CRT-D	33 (61.1%) * 21 (38.9%) *
Number of catheters 1 2 3 4	31 (26.7%) 55 (47.4%) 22 (19%) 8 (6.9%)
Number of extracted catheters 0 1 2 3 4	3 (2.6%) 36 (31%) 51 (44%) 18 (15.5%) 8 (6.9%)
Previously abandoned catheter ≥1	14 (12.1%)
Technique of extraction Traction Mechanical dilator sheath Powered sheath	9 (7.56%) 93 (78.15%) 13 (10.9%)
Procedure duration, minutes	129 ± 50.2
Procedural success complete clinical surgical extraction procedural failure complications	101 (84.9%) 8 (6.7%) 2 (1.7%) 1 (0.8%) 7 (5.9%)
Follow up, months	49 (48)

**Table 2:** Study population stratified by diagnosis of infection.

Variable	Infected (n = 82)	Non Infected (n = 37)	*p*-Value
Male sex	65 (79.3%)	27 (73%)	0.45
Coronary artery disease	24 (30%)	14 (38%)	0.60
Heart failure	33 (43.4%)	23 (65.7%)	0.03
Atrial fibrillation	27 (36%)	13 (37%)	0.91
Systemic arterial hypertension	54 (71%)	20 (59%)	0.21
Diabetes	18 (23.7%)	6 (17.6%)	0.48
Anemia	17 (22.4%)	7 (20.6%)	0.89
White blood cells <4500 (leukopenia) 4500–9800(normal range) >9800 (leukocytosis)	26 (35%) 40 (53%) 9 (12%)	11 (33%) 20 (61%) 2 (6%)	0.68
C-reactive protein ≥3 mg/dL	57 (81%)	14(50%)	0.002
Creatinine, mg/dL	1.1 (0.65)	1.1 (0.4)	0.25
Chronic kidney disease	23 (30.6%)	8 (24.2%)	0.49
Left ventricular ejection fraction <30% 30–50% >50%	11 (18%) 25 (41%) 25 (41%)	5 (19.2%) 14 (53.8%) 7 (27%)	0.43
Age at extraction, years	79.7 (12)	68.3 (20.8)	<0.001
Number of implants 1 2 3 4	32 (58.2%) 15 (27.3%) 6 (10.9%) 2 (3.6%)	14 (66.6%) 4 (19%) 3 (14.2%) 0 (0%)	0.84
Older leads, months	109 (82)	66 (65.6)	0.03
Type of device Single PM Single ICD Dual PM Dual ICD CRT-P CRT-D	3 (3.8%) 9 (11.2%) 36 (45%) 6 (7.5%) 10 (12.5%) 16 (20%)	1 (2.9%) 13 (37.1%) 6 (17.1%) 9 (25.7%) 2 (5.8%) 4 (11.4%)	<0.001
Coil (yes/no)	31 (38.75%)	26 (74.2%)	<0.001
Coil Single Dual * out of 58 pts implanted with single ICD, dual ICD, CRT-D	15 (51.7%) * 14 (48.2%) *	18 (72%) * 7 (28%) *	0.13
Number of catheters 1 2 3 4	13 44 16 8	18 11 6 0	<0.001
Previously abandoned catheter ≥1	13 (16%)	1 (2.9%)	0.06

**Table 3:** Procedural Characteristics stratified by diagnosis of infection.

Variable	Infected (n = 82)	Non Infected (n = 37)	*p*-Value
Procedure duration, minutes	125 ± 47.4	137 ± 55.6	0.22
Number of extracted catheters 0 1 2 3 4	- 14 (17.3%) 42 (51.9%) 17 (21%) 8 (9.8%)	3 (8.6%) 22 (62.9%) 9 (25.7%) 1 (2.8%) -	<0.001
Previously abandoned catheter ≥1	13 (16%)	1 (2.9%)	0.06
Technique of extraction Traction Mechanical dilator sheath Powered sheath	6 (7.4%) 64 (79%) 10 (13.6%)	3 (8.6%) 29 (82.8%) 3 (8.6%)	0.90
Procedural success complete clinical surgical extraction procedural failure complications	72 (87.8%) 4 (4.9%) 1 (1.2%) 0 (0%) 5 (6.1%)	29 (78.4%) 4 (10.8%) 1 (2.7%) 1 (2.7%) 2 (5.4%)	0.30

**Table 4:** Univariate and multivariate Cox regression for all-cause death.

	Univariable		Multivariable	
Variable	HR (95% CI)	*p*-Value	HR (95% CI)	*p*-Value
Infective indication	1.46 (0.76–2.78)	0.25		
Age at extraction	1.03 (1.01–1.06)	0.009	1.03 (1.00–1.06)	0.05
Coronary artery disease	1.71 (0.94–3.10)	0.08		
Atrial fibrillation	2.81 (1.56–5.07)	0.001	2.54 (1.37–4.72)	0.003
Systemic arterial hypertension	0.84 (0.47–1.53)	0.58		
Diabetes	1.66 (0.87–3.17)	0.12		
Anemia	2.02 (1.1–3.73)	0.05	1.9 (0.99–3.64)	0.009
Chronic kidney disease	1.63 (0.90–2.96)	0.11	1.78 (1.10–2.86)	0.02
Left ventricular ejection fraction	0.99 (0.96–1.02)	0.58		
Heart failure	0.88 (0.49–1.58)	0.65		
Presence of coil	0.70 (0.40–1.23)	0.22		
Multiple leads (n ≥ 2)	1.42 (0.73–2.76)	0.30		
Older leads	1.00 (1.00–1.01)	0.41		

## Data Availability

The data presented in the study are available on request from the corresponding author.

## Supplementary Materials

The following supporting information can be downloaded at: https://www.mdpi.com/article/10.3390/jcm12134543/s1, Table S1 Study population characteristics stratified by diagnosis of local versus systemic infection.
