# Supplementary material for: Long-Term Outcomes of Transvenous Lead Extraction: A Comparison in Patients with or without Infection from the Italian Region with the Oldest Population
Source: J Clin Med. 2023 Jul 7;12(13):4543. doi: 10.3390/jcm12134543 (PMC10342464; doi:10.3390/jcm12134543)
Supplement: Supplementary file 1 [file jcm-12-04543-s001.zip › jcm-2447210-supplementary.pdf]

## Supplementary data

**Table S1.** Study population characteristics stratified by diagnosis of local versus systemic infection.

| Variable | Local Infection (n=67) | Systemic infection (n= 15) | p-value |
|---|---|---|---|
| Male sex | 54 (80.5%) | 11 (73%) | 0.50 |
| Coronary artery disease | 21 (34.4%) | 5 (35.7%) | 1 |
| Heart failure | 27 (43.5%) | 6 (43%) | 1 |
| Atrial Fibrillation | 21 (34.4%) | 6 (43%) | 0.55 |
| Systemic arterial hypertension | 45 (72.5%) | 9 (64%) | 0.53 |
| Diabetes | 15 (24.2%) | 3 (21.4%) | 1 |
| Anemia | 10 (16.4%) | 7 (46.7%) | **0.012** |
| White blood cells | | | 0.51 |
| *<4500 (leukopenia)* | 22 (36.6%) | 4 (26.6%) | |
| *4500-9800(normal range)* | 32 (53.3%) | 8 (53.2%) | |
| *>9800 (leukocytosis)* | 6 (11.1%) | 3 (20.2%) | |
| C-reactive protein ≥3 mg/dL | 44 (78.5%) | 13(92.8%) | 0.44 |
| Chronic kidney disease | 16 (23.88%) | 7 (46.67%) | 0.13 |
| Left ventricular ejection fraction | | | 0.60 |
| *<30%* | 21 (43.8%) | 4 (30.8%) | |
| *30-50%* | 9 (18.7%) | 2 (15.4%) | |
| *>50%* | 18 (37.5%) | 7 (53.8%) | |
| Age at extraction, years | 79.7 [11.2] | 78.7 [12.3] | 0.91 |
| Number of implants | | | 0.92 |
| *1* | 26 (59.1%) | 6 (54.4%) | |
| *2* | 11 (25%) | 4 (36.6%) | |
| *3* | 5 (11.4%) | 1 (9%) | |
| *4* | 2 (4.5%) | 0 (0%) | |
| Older leads, months | 109 [82] | 112 [76] | 0.98 |
| Type of device | | | 0.31 |
| *Single PM* | 2 (3.1%) | 1 (6.6%) | |
| *Single ICD* | 7 (10.8%) | 2 (13.4%) | |
| *Dual PM* | 31 (47.7%) | 5 (33.3%) | |
| *Dual ICD* | 3 (4.6%) | 3 (20%) | |
| *CRT-P* | 9 (13.8%) | 1 (6.7%) | |
| *CRT-D* | 13 (20%) | 3 (20%) | |
| Coil (yes/no) | 23 (35.4%) | 8 (53.3%) | 0.24 |
| Coil | | | 0.68 |
| *Single* | 10 (47.6%)* | 5 (62.5%)* | |
| *Dual* | 11 (52.4%)* | 3 (37.5%)* | |
| **out of 31 pts implanted with single ICD, dual ICD, CRT-D* | | | |
| Number of catheters | | | 0.14 |
| *1* | 8 (12.1%) | 5 (33.3%) | |
| *2* | 37 (56.1%) | 7 (46.7%) | |
| *3* | 15 (22.7%) | 1 (6.7%) | |
| *4* | 6 (9.1%) | 2 (13.3%) | |
| Number of extracted catheters | | | 0.17 |
| *1* | 9 (13.6%) | 5 (33.3%) | |
| *2* | 35 (53%) | 7 (46.7%) | |
| *3* | 16 (24.4%) | 1 (6.6%) | |
| *4* | 6 (9%) | 2 (13.4%) | |
| Previously abandoned catheter ≥1 | 10 (17.85%) | 3 (20%) | 0.70 |
| Technique of extraction | | | 0.53 |
| *Traction* | 6 (%) | 0 (0%) | |
| *Mechanical dilator sheath* | 51 (%) | 14 (93.3%) | |
| *Powered sheath* | 9 (%) | 1 (6.7%) | |
| Procedure duration, minutes | 127 ± 45.1 | 115 ± 57 | 0.38 |

| | | | |
|---|---|---|---|
| Procedural success | | | 0.22 |
| *complete* | 60 (89.6%) | 12 (80%) | |
| *clinical* | 3 (4.4%) | 1 (6.7%) | |
| *surgical extraction* | 0 (0%) | 1 (6.7%) | |
| *procedural failure* | 0 (0%) | 0 (0%) | |
| *complications* | 4 (6%) | 1 (6.7%) | |
